# Recent and current developments in handling Markush structures from chemical patents

**DOI:** 10.1186/1758-2946-4-S1-O18

**Published:** 2012-05-01

**Authors:** John M Barnard, Geoff M Downs

**Affiliations:** 1 Digital Chemistry Ltd., Sheffield S26 1HL, UK; 2 Digital Chemistry Ltd., Sheffield S26 1HL, UK

## 

The commercially-available database systems for storing and searching Markush structures from chemical patents have undergone little change since their launch some twenty years ago. However, the past few years have seen the area become an active one again for research and development. This presentation offers an overview and commentary on recent and current activity, and discusses the prospects for improved access to structural information in the patent literature [1].

The existing curated Markush databases remain the gold standard, though several groups, both academic and commercial, continue to work on automatic analysis of full-text patents. This has involved not only the identification of specific-structure nomenclature and its conversion to structure-searchable records, but also the attempted reconstruction of searchable representations of complete Markush structures. The advantages and disadvantages of these approaches, and the prospects for their successful commercial exploitation, are discussed.

New commercial software for searching Markush structure databases is being developed by several groups. These employ both conventional substructure search approaches (e.g. ChemAxon, Digital Chemistry), and novel algorithms, in some cases based on various forms of similarity and approximate structure matching (e.g. DecrIPt, IBM). These approaches are summarised and compared, and the opportunity their in-house implementation provides for integration of chemical patent information with the drug-discovery process is discussed.

Practitioners have long been aware of the inadequacies and complexities of the existing systems, and the extent to which a new generation of systems may satisfy their requirements is discussed. The possible role of systematic evaluation of retrieval performance (in particular, the TREC-CHEM project) is addressed.
